# A Homozygous Nonsense Thyroid Peroxidase Mutation (R540X) Consistently Causes Congenital Hypothyroidism in Two Siblings Born to a Consanguineous Family

**DOI:** 10.4274/jcrpe.1920

**Published:** 2015-12-03

**Authors:** Hakan Cangül, Murat Doğan, Duran Üstek

**Affiliations:** 1 Medipol University International Faculty of Medicine, Department of Medical Genetics, İstanbul, Turkey; 2 Yüzüncü Yıl University Faculty of Medicine, Division of Pediatric Endocrinology, Van, Turkey

**Keywords:** thyroid peroxidase, gene, mutation, genetics, molecular, congenital hypothyroidism, thyroid dyshormonogenesis

## Abstract

**Objective:**

Congenital hypothyroidism (CH) is the most common neonatal endocrine disorder, and mutations in the thyroid peroxidase (TPO) gene have been reported to cause the disease. Our aim in this study was to determine the genetic basis of CH in two affected children coming from a consanguineous family.

**Methods:**

First, we investigated the potential genetic linkage of the family to any known CH locus using microsatellite markers and then screened for mutations in the linked gene by Sanger sequencing. By using next-generation sequencing, we also checked if any other mutation was present in the remaining 10 causative CH genes.

**Results:**

The family showed potential linkage to the TPO gene, and we detected a homozygous nonsense mutation (R540X) in both cases. The two patients had total iodide organification defect (TIOD). Both the microsatellite marker haplotypes and the mutation segregated with the disease status in the family, i.e. all healthy subjects were either heterozygous carriers or homozygous wild-type, confirming the pathogenic nature of the mutation. Neither was the mutation present in any of the 400 control chromosomes nor were there any other mutations in the remaining causative CH genes.

**Conclusion:**

This study proves the pathogenicity of R540X mutation and demonstrates the strong genotype/phenotype correlation associated with this mutation. It also highlights the power of working with familial cases in revealing the molecular basis of CH and in establishing accurate genotype/phenotype relationships associated with disease causing mutations.

**WHAT IS ALREADY KNOWN ON THIS TOPIC?**

Thyroid peroxidase (TPO) mutations cause congenital hypothyroidism.

**WHAT THIS STUDY ADDS?**

R540X mutation in the TPO gene is pathogenic and associated with total iodide organification defect.

## INTRODUCTION

Congenital hypothyroidism (CH) is the most common inborn error of metabolism in newborns and leads to mental retardation unless recognised and intervened early (1). The disease originates from congenital deficiency of thyroid hormones due to a developmental problem in the thyroid gland or a defect in thyroid hormone bio-synthesis. Both situations might result from germ-line mutations in the genes encoding components of these pathways, leading to the precipitation of disease in familial cases (2). CH is genetically heterogeneous, and causative genes discovered for the development of disease are divided into two main groups: (i) genes involved in the development of the thyroid gland (3,4) and (ii) genes involved in thyroid hormone bio-synthesis (5). Loss-of-function (LOF) mutations of the genes involved in gland development cause thyroid dysgenesis, while LOF mutations of the genes involved in hormone synthesis lead to thyroid dyshormonogenesis (TDH). TDH is mostly inherited autosomal recessively in familial cases. Seven genes have been described to date, mutations in which cause TDH (6). These genes encode enzymes and transporters that are involved in organification of iodide into the thyroid gland and following steps in thyroid hormone synthesis (7).

Thyroid peroxidase (TPO) is one of these genes, and LOF mutations in this gene result in TDH (8). The protein product of this gene, TPO enzyme, catalyses both oxidation and organification of iodide by using hydrogen peroxide (H2O2) as the final electron acceptor (8,9,10). Defects in H2O2 generation are associated with CH (11,12,13). DUOX1 and DUOX2 enzymes are crucial for H2O2 generation (14,15). TPO enzyme is located in the apical membrane of the thyroid cell where it catalyses oxidation and organification of iodide. Moreover, it also catalyses coupling reactions of monoiodotyrosine and diiodotyrosines into thyroid hormones free thyroxine (fT4) and free triiodothyronine (fT3) (2). Germline LOF mutations in the TPO gene are associated with primary CH and TDH, and currently 60 mutations have been reported in this gene (8,9).

To investigate the genetic background of CH, we developed a two-tier strategy combining genetic linkage studies and full sequencing of candidate genes in familial cases and have identified several mutations to date in different CH genes (16,17,18,19,20,21,22,23,24,25,26,27,28,29,30). In the current study, we aimed to determine the genetic cause of CH in a consanguineous family with two affected siblings. Here, we report a detailed genetic analyses and associated clinical phenotypes of these cases. Molecular genetic analyses facilitate definitive diagnosis and accurate classification of CH and might also describe patient-specific targets for alternative treatment of the disease.

## METHODS

### Subjects

Two cases born to a consanguineous Turkish family were ascertained through our studies on the genetics of CH (16,17,18,19,20,21,22,23,24,25,26,27,28,29,30). The older sister was first diagnosed at age six months. At this time, her hormone values were: thyroid-stimulating hormone (TSH) 150 mIU/L (normal 0.3-5), T4 <4.5 µg/dL (normal 4.5-12.5), and T3 <80 ng/dL (normal 80-120). Thyroid ultrasonoghraphy and scintigraphy showed thyroid hyperplasia, and perchlorate discharge test indicated total iodide organification defect (TIOD). In her last follow-up at the age of 12, she was on 100 µg/day L-T4 and her hormone levels were TSH 1.82 mIU/L and fT4 1.40 ng/dL. Her weight was <3rd centile and height was at 25th centile.

The younger brother was born at term and first diagnosed at age 40 days when he underwent a thorough investigation for prolonged jaundice. Hormone values at diagnosis were TSH 190 mIU/L, T4 1.5 µg/dL (normal 4.5-12.5), and T3 20 ng/dL (normal 80-120). Thyroid ultrasonography performed at age 3 months showed diffuse hyperplasia, while a scintigraphy at age 4 years demonstrated hyperplasia with homogeneous dissemination of activity. Perchlorate discharge test indicated TIOD. At the age of 10 years, he has mild mental retardation and his weight is under 10th centile and height at 3rd centile.

The parents were healthy and free of any signs or symptoms of hypothyroidism. Informed consent was obtained from the family, and venous blood samples were collected from all family members. All procedures performed were in accordance with the Declaration of Helsinki, and the study was approved by relevant IRBs/Ethics Committees. DNA was extracted by using standard methods and stored at -20 °C until analysed.

### Potential Linkage Analysis

First, we performed linkage analysis to all 11 known CH loci in all family members with the use of microsatellite markers. Four primer pairs surrounding each locus were selected (Table 1). Fluorescent labelling of one oligonucleotide of each primer pair enabled the sizing of polymerase chain reaction (PCR) products in a capillary electrophoresis machine by using Gene Mapper v4.0 software suite (Applied Biosystems, Warrington, UK). By combining genotypes for microsatellite markers, we constructed haplotype tables for each family member. As autosomal recessive inheritance was assumed in consanguineous families, homozygosity of a particular haplotype for a locus in cases accompanied by heterozygosity of the same haplotype in both parents was taken as suggestive of linkage to that locus.

### Direct Sequence Analysis of the Thyroid Peroxidase Gene

The DNA template of the TPO gene was downloaded from the Ensembl database (ENSG00000115705). All alternative transcripts (17 in total) were included to ensure that primers were designed to cover all coding exons and intron/exon boundaries. Intronic primers flanking the coding sequence were designed for PCR amplification using Exon Primer and Primer 3. Primer sequences and PCR conditions are available upon request. PCR products were size-checked on 1% horizontal agarose gels and cleaned up using MicroCLEAN (Microzone, Haywards Heath, UK) or gel-extracted using QIAquickTM Gel Extraction kit (Qiagen, Crawley, UK). The purified PCR products were sequenced in both forward and reverse directions using the ABI BigDye Terminator v3.1 Cycle Sequencing kits on an ABI Prism 3730 DNA Analyzer (Applied Biosystems, Warrington, UK). Analysed sequences were then downloaded using Chromas software and assessed for the presence of alterations.

### Next-Generation Sequencing for Mutation Screening in Other Causative Congenital Hypothyroidism Genes

We chose Illumina’s TruSeq Custom Amplicon (TSCA) assay for next-generation sequencing (NGS) library preparation (Burrington, UK). Illumina Design Studio (http://designstudio.illumina.com/) was used to create the optimal TSCA panel design for 16 selected CH genes, 11 known-causative (Table 1), and 5 of our own candidates. Prior to target enrichment, the selected samples were diluted to 25 ng/μL, with calculations based on average DNA concentration from duplicate Qubit® assays. Samples of concentration <25 ng/μL (but ≥16.7 ng/μL) were used neat, with volume increased from the standard 10 μL up to a maximum of 15 μL (such that the recommended 250 ng DNA was added, as for all samples). Target enrichment was performed using the designed TSCA kit in accordance with manufacturer’s instructions (Illumina TruSeq® Custom Amplicon Library Preparation Guide. Part #15027983). TSCA libraries were sequenced on the in-house Illumina MiSeq platform using a 500 cycle reagent kit and paired-end sequencing (2x251 cycle reads). In accordance with manufacturer’s instructions (Illumina MiSeq System User Guide. Part #15027617), the diluted amplicon library was transferred into the reagent cartridge, and this was loaded onto the MiSeq alongside the Illumina-provided flowcell and buffer. Initial data analysis was automatically performed on-instrument (MiSeq Reporter software, TruSeq Amplicon Workflow), which demultiplexes indexed reads, generates FASTQ files, and performs alignment. Analysis of coverage and variant calling was performed using SoftGenetics NextGENe v2.3.3. For the positive controls, NextGENe’s Mutation Report and sequence alignment view were used to examine calling and coverage of all known variants. Finally, NextGENe’s Variant Comparison Tool was used for each run to generate a list of all called variants across samples.

## RESULTS

**Figure 1:**
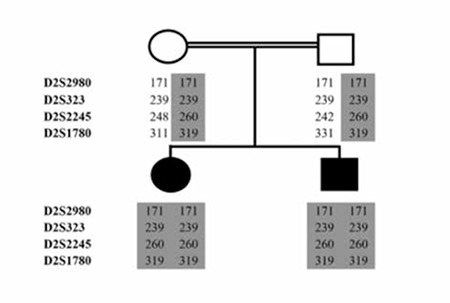
The scores of microsatellite marker analysis surrounding the TPO locus depicted on the family pedigree. The markers used are listed on the left and the disease-associated haplotype is shaded in grey

Haplotype tables were constructed for each family member by combining the scores for each marker to observe the segregation of the genotype along with the disease status. The linkage analysis using these tables indicated a potential linkage to the TPO locus in the family, i.e. both CH cases were homozygous for a disease-associated haplotype, while both parents were heterozygous for the same haplotype (Figure 1). The family showed no such linkage to other 10 loci tested, i.e. the cases did not have homozygosity for the microsatellite markers designed for these loci. These results suggested that the disease-associated haplotype segregated with the disease status in the family assuming autosomal recessive inheritance model which is the most likely pattern in consanguineous families, i.e. the cases were homozygous and all unaffected members of the family were heterozygous for the haplotype. Therefore, we proceeded to sequence the entire coding region (and flanking sequences) of the TPO gene in all family members.

**Figure 2:**
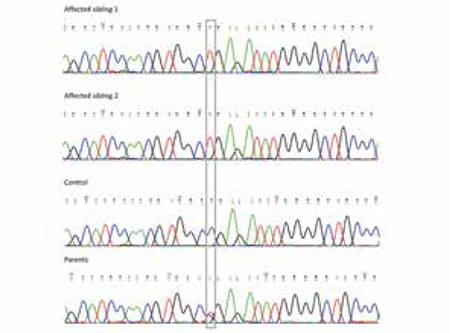
Sequence traces from the TPO gene showing the mutation R540X (c.1618C>T, in grey rectangle, numbering according to Ensembl transcript ENST00000329066 where ATG is taken as the transcription start site). Note the homozygosity of the mutation in the cases (top panels) and heterozygosity in the parents (bottom panel) compared to the control (middle panel)

Direct sequencing of the TPO gene revealed a homozygous C to T (c.1618C>T) in both affected siblings which results in a stop codon leading to truncation of the enzyme molecule (p.R540X). Both parents carried the mutation at heterozygous state (Figure 2) which was consistent with the linkage data. These results indicated the proper segregation of the mutation with the disease status in the family according to an autosomal recessive inheritance pattern as expected in consanguineous families. Mutation analysis in the rest of the TPO sequence revealed no other mutations in the family. The mutation was not present in 400 ethnically-matched control chromosomes. The results of the mutation screening by NGS indicated that neither case nor the other family members carried any mutation in the remaining causative CH genes.

## DISCUSSION

**Figure 3:**
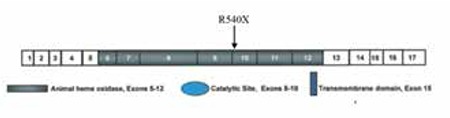
Schematic view of the human TPO structure showing the location of the R540X mutation. The truncation of the molecule at 540th residue by this mutation will result in lack of important catalytic domains in the subsequent enzyme if expressed at all

The TPO gene is located on chromosome 2p25 and covers about 150 Kb of DNA (1,19). It is comprised of 17 coding exons with a 3048 nucleotide-full-length transcript which encodes 933 amino acid-TPO enzymes. C to T transitions are among the most common genetic alterations leading to early stop codons in genes and to truncation of resulting gene products. The transition detected in this study (c.1618C>T) leads to a stop codon in the TPO gene and the truncation of the resulting enzyme molecule at 540th residue (R540X). This truncated enzyme will lack important catalytic domains (Figure 3), if expressed at all as nonsense mutations located before the last exon of any gene is expected to trigger nonsense-mediated decay of transcripts resulting with no protein expression (31). Therefore, the R540X mutation detected in this study is expected to lead to total lack of enzyme activity in our patients. However, as thyroid tissues of the subjects were not available, it was not possible to show this in vivo.

In terms of phenotypic presentation, both siblings in our study showed TIOD indicating an extensive loss in TPO activity. In their comprehensive review (8), Ris-Stalpers and Bikker stated that homozygous or compound heterozygous inactivating TPO mutations are associated with goitre and TIOD. As some missense mutations might result in partial loss of function in the TPO enzyme with some residual activity, they may be manifested as partial iodide organification defects. Clinical presentation in cases with these mutations could be further modified with iodine status. Therefore, the phenotypes in those cases are more likely to be variable. In contrast, total LOF mutations like Y540X invariably lead to TIOD and thus allow the establishment of more reliable genotype/phenotype correlations. Accordingly, it is fair to suggest that the Y540X mutation detected in this study is phenotypically associated with severe TDH and TIOD.

In this study, we presented several lines of evidence for the pathogenicity of the R540X mutation in the TPO gene: (i) homozygosity of the mutation in the case; (ii) proper segregation of both the mutation and the microsatellite marker haplotypes with the disease status in the family according to autosomal recessive inheritance pattern; (iii) absence of any other mutation of the TPO gene in the cases; (iv) absence of any mutation in all other known causative CH genes in the family; (v) absence of the mutation in 400 ethnically-matched control chromosomes; (vi) severe CH phenotype in the cases as expected from the functional effect of the mutation on the resulting TPO enzyme.

Therefore, this study clearly proves that R540X in the TPO gene is pathogenic and causes CH in our cases. Additionally, it also demonstrates that this mutation is associated with severe CH phenotype and leads to TIOD in the subjects. Once again, this study highlights the crucial role of genetic investigations in familial cases for revealing the molecular basis of CH as well as for establishing accurate genotype/phenotype relationships associated with disease-causing mutations.

## Figures and Tables

**Table 1:**
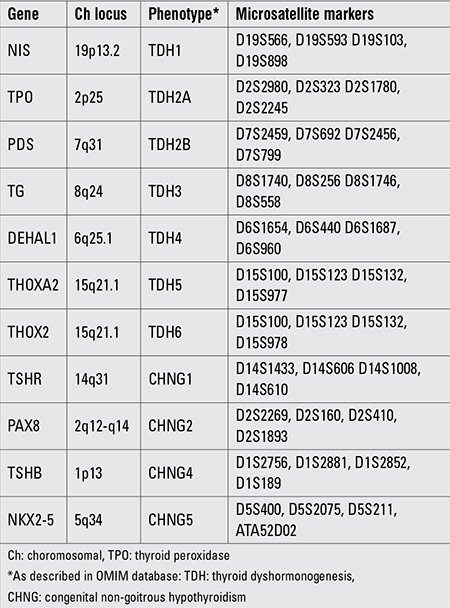
Genes causing congenital hypothyroidism, associated phenotypes, and microsatellite markers used for their linkage analysis
